# Intratumoral delivery of TransCon^™^ TLR7/8 Agonist promotes sustained anti-tumor activity and local immune cell activation while minimizing systemic cytokine induction

**DOI:** 10.1186/s12935-022-02708-6

**Published:** 2022-09-19

**Authors:** Luis Alejandro Zúñiga, Torben Leßmann, Karan Uppal, Nicola Bisek, Enping Hong, Caroline E. Rasmussen, Jens-Jakob Karlsson, Joachim Zettler, Lars Holten-Andersen, Kathy Bang, Dhruv Thakar, Yu-Chi Lee, Salomon Martinez, Simran Singh Sabharwal, Sebastian Stark, Frank Faltinger, Oliver Kracker, Samuel Weisbrod, Robin Müller, Tobias Voigt, Kornelia Bigott, Mohammad Tabrizifard, Vibeke Miller Breinholt, Amer M. Mirza, David B. Rosen, Kennett Sprogøe, Juha Punnonen

**Affiliations:** 1 Ascendis Pharma, Inc., Redwood City, CA USA; 2 Ascendis Pharma GmbH, Heidelberg, Germany; 3 Ascendis Pharma A/S, Hellerup, Copenhagen, Denmark

**Keywords:** Toll-like receptor, Tumor microenvironment, Innate immunity, Adaptive immunity, Intratumoral treatment, Intratumoral injection

## Abstract

**Background:**

Intratumoral (IT) delivery of toll-like receptor (TLR) agonists has shown encouraging anti-tumor benefit in preclinical and early clinical studies. However, IT delivery of TLR agonists may lead to rapid effusion from the tumor microenvironment (TME), potentially limiting the duration of local inflammation and increasing the risk of systemic adverse events.

**Methods:**

To address these limitations, TransCon^™^ TLR7/8 Agonist—an investigational sustained-release prodrug of resiquimod that uses a TransCon linker and hydrogel technology to achieve sustained and predictable IT release of resiquimod—was developed. TransCon TLR7/8 Agonist was characterized for resiquimod release in vitro and in vivo, in mice and rats, and was assessed for anti-tumor efficacy and pharmacodynamic activity in mice.

**Results:**

Following a single IT dose, TransCon TLR7/8 Agonist mediated potent tumor growth inhibition which was associated with sustained resiquimod release over several weeks with minimal induction of systemic cytokines. TransCon TLR7/8 Agonist monotherapy promoted activation of antigen-presenting cells in the TME and tumor-draining lymph nodes, with evidence of activation and expansion of CD8^+^ T cells in the tumor-draining lymph node and TME. Combination of TransCon TLR7/8 Agonist with systemic immunotherapy further promoted anti-tumor activity in TransCon TLR7/8 Agonist-treated tumors. In a bilateral tumor setting, combination of TransCon TLR7/8 Agonist with systemic IL-2 potentiated tumor growth inhibition in both injected and non-injected tumors and conferred protection against tumor rechallenge following complete regressions.

**Conclusions:**

Our findings show that a single dose of TransCon TLR7/8 Agonist can mediate sustained local release of resiquimod in the TME and promote potent anti-tumor effects as monotherapy and in combination with systemic immunotherapy, supporting TransCon TLR7/8 Agonist as a novel intratumoral TLR agonist for cancer therapy. A clinical trial to evaluate the safety and efficacy of TransCon TLR7/8 Agonist, as monotherapy and in combination with pembrolizumab, in cancer patients is currently ongoing (transcendIT-101; NCT04799054).

**Supplementary Information:**

The online version contains supplementary material available at 10.1186/s12935-022-02708-6.

## Background

Toll-like receptors (TLRs) are a family of evolutionarily conserved pattern recognition receptors that play a critical role in activating both innate and adaptive immune cells, making them potential targets in oncology for harnessing the body’s immune system in killing tumor cells. TLRs bind pathogen and malignant cell-derived ligands which trigger the NF-κB and interferon regulatory factor pathways, resulting in the production of proinflammatory cytokines, chemokines, and expression of immune stimulatory molecules by innate immune cells and antigen-presenting cells (APCs), such as dendritic cells [1]. TLR activation induces APC maturation, thus enhancing antigen uptake/presentation and activation of effector T cells. Furthermore, TLR activation promotes immune cell recruitment and can modulate regulatory T cell activity [2–4]. These mechanisms of innate immune activation make TLR agonists attractive molecules for cancer therapy alone or in combination with other complementary immunotherapies that directly increase the function of cytotoxic immune cells [5].

While several TLR agonists are being developed for cancer therapy, only one is approved thus far. Imiquimod, a small molecule TLR7 agonist, is FDA approved as a topical formulation for basal cell carcinoma, genital warts, and actinic keratoses [6]. Resiquimod, also known as R848, is a small molecule agonist of both TLR7 and TLR8 that has been evaluated using a topical formulation in clinical trials for the treatment of cutaneous T cell lymphoma (CTCL) [7]. In CTCL, when lesions were treated topically with a 0.03% or 0.06% resiquimod gel, significant improvement of lesion clearance was observed in 75% of patients, with only minor adverse events (AEs) related to skin irritation [7]. These clinical data suggest that local resiquimod application has potential therapeutic utility with an acceptable safety profile.

Direct intratumoral (IT) delivery of innate immunostimulators, including TLR agonists, has shown robust anti-tumor activity in preclinical models [8–10]. Additionally, clinical studies using local delivery of TLR7 [11], TLR7/8 [12], and TLR9 agonists [13–15] have demonstrated encouraging early evidence of clinical anti-tumor efficacy. Despite the promise of IT delivery of innate immune modulators for promoting anti-tumor immunity, the safety and efficacy of the IT route of administration has suffered from rapid effusion of administered compounds from the injection site, which necessitates frequent dosing regimens, and may result in systemic exposure of active compounds, leading to undesired systemic proinflammatory cytokine induction [16–19].

TransCon (Transient Conjugation) technology was developed to temporarily link a parent compound to a soluble or insoluble carrier to enable sustained release of parent compounds for either systemic or local applications. TransCon technology has been applied to create a subcutaneously administered, sustained release prodrug of human growth hormone that was FDA [20] and EMA [21] approved as a once weekly therapy for children with growth hormone deficiency. TransCon technology has also been applied to create sustained release prodrugs of human parathyroid hormone (TransCon PTH) for hypoparathyroidism and of C-type natriuretic peptide (TransCon CNP) for achondroplasia, both of which are currently in late-stage clinical development. Using TransCon technology, TransCon TLR7/8 Agonist was designed to address the current limitations of TLR agonists and IT delivery through sustained local release of resiquimod. Specifically, to create TransCon TLR7/8 Agonist, resiquimod was conjugated to degradable polymeric hydrogel microspheres via a TransCon linker—a covalent but cleavable linkage—allowing for predictable, sustained release of resiquimod under control of the physicochemical environment. This approach is expected to minimize systemic drug exposure and promote several anti-tumor immune mechanisms such as (1) activation and maturation of APCs [22], (2) expression of type-I and type-II interferons associated with anti-tumor immunity [23], (3) migration of activated APCs to tumor draining lymph nodes (tDLNs) [24], and (4) activation and recruitment of cytotoxic immune cell subsets to the tumor microenvironment (TME) [8].

A single IT administration of TransCon TLR7/8 Agonist was sufficient to provide sustained exposure of resiquimod over several weeks, with minimal systemic cytokine release. Significant single agent anti-tumor activity was observed, and cytokine and immune cell profiling provided evidence of prolonged activation of innate and adaptive immune cells in tumor and tumor-draining lymph nodes, with evidence of an increase in the frequency of tumor-antigen specific CD8^+^ T cells in the TME. Finally, combining a single dose of TransCon TLR7/8 Agonist with systemic immunotherapy enhanced tumor growth inhibition in both injected and non-injected tumors, and demonstrated immune memory in a tumor rechallenge setting. Altogether, these data characterize TransCon TLR7/8 Agonist as a novel TLR therapy with the potential to provide clinical benefit to patients with cancer.

## Materials and methods

### Chemicals/test items

Resiquimod was obtained from abcr (Karlsruhe, Baden-Württemberg) or Fluorochem (Hadfield, Derbyshire). Empty (TransCon Vehicle) and TransCon TLR7/8 Agonist hydrogels were prepared per methods described in detail in patent application US 20220062273 A1. Buffer control consisted of a phosphate-based buffer.

### In vitro resiquimod quantification

For in vitro assessment of resiquimod release from TransCon TLR7/8 Agonist, a suspension of TransCon TLR7/8 Agonist was incubated in 60 mM phosphate buffer at 37 °C at various pH levels. Samples of the supernatant were withdrawn at various times and resiquimod content was quantified by liquid chromatography and compared to the initial amount. Release half-lives were determined following a first-order fit of the data (GraphPad Prism 7.05).

### Pharmacokinetic studies

For plasma resiquimod quantification, resiquimod (10 mM succinate, 90.0 mg/ml trehalose dihydrate, pH 5.0 at a concentration of 104 µg/ml) or TransCon TLR7/8 Agonist [suspended (ca. 6% wt/v) in PBST buffer at pH 7.4] was injected SC into rats or IT into mice bearing single CT26 syngeneic tumors and plasma levels of resiquimod were observed over time. Male WISTAR rats (n = 3 per group) received a single SC injection of resiquimod solution or TransCon TLR7/8 Agonist, each corresponding to a dose of 25 µg eq. of resiquimod. Female CT26 tumor bearing mice (n = 3 per time point) received a single intratumoral injection of TransCon TLR7/8 Agonist corresponding to a dose of 5 or 20 µg eq. of resiquimod. Blood was withdrawn (n = 3 mice per time point) and used for plasma generation at 6 h, 3 days and 7 days. Resiquimod concentration in plasma samples was quantified via liquid chromatography with tandem mass spectrometry (LC–MS/MS) with selected MRM transitions using a deuterated internal standard and solid-phase extraction method for sample preparation. Plasma concentration profiles were generated and analyzed with Phoenix WinNonlin software (Certara, Princeton, NJ, USA).

### Rodents

All animal experiments were carried out following local government and institutional guidelines and regulations. All in vivo protocols were approved by a local Institutional Animal Care and Use Committee, or other appropriate review board, before study execution. All animals were housed in accordance with local regulations. Animal studies were performed by Charles River Discovery Research Services Germany GmbH (Freiburg, BW, Germany), Charles River Discovery Services (Morrisville, NC, USA), Crown Bioscience Inc. (Taicang, JS, China), Heidelberg Pharma AG (Ladenburg, BW, Germany), or at Ascendis Pharma, Inc. (Redwood City, CA, USA). Feed and water were provided ad libitum. Sample collection and euthanasia were performed according to all relevant animal welfare guidelines.

### Cell culture

Tumor cells were maintained in vitro with RPMI-1640 medium with 10% fetal bovine serum (CT26) or Dulbecco’s Modified Eagle Medium (DMEM) with 10% fetal bovine serum (MC38) and kept at 37 °C in an atmosphere of 5% CO_2_ in air. Cells in exponential growth phase were harvested and cell number/viability were quantitated by cell counter before tumor inoculation.

### In vivo tumor models

For CT26 tumor experiments, 6- to 10-week-old female BALB/c mice were SC inoculated with 0.3–2 × 10^6^ CT26 cells on the right (single tumor bearing) or both flanks as indicated. For syngeneic MC38 tumor experiments, 8-week-old female C57BL/6NCrl mice were SC inoculated with 0.5 × 10^6^ MC38 cells on their right flank. For tumor rechallenge, mice that experienced a complete regression in both treated and non-treated primary tumors for at least 10 days were SC injected with 5 × 10^5^ CT26 cells in their front right dorsal region and observed for tumor growth.

Tumor diameter was measured with calipers and tumor volumes were calculated following the formula: tumor volume = (L × W^2^) × 0.5 where L is length of the tumor and W the width (both in mm). Mice were randomly enrolled into treatment groups when tumors reached an average volume of ~ 80–200 mm^3^, aiming at comparable group median and mean tumor volumes either using a stratified randomization or Matched Distribution method. Study-specific tumor enrollment criteria and group sizes are indicated in the figure legends. The enrollment day is denoted as day 0 (D0). Tumor volumes, body weight, and body condition score [25] were recorded 2–3 times every week. Experimenters were not blind to group assignment and outcome assessment.

A 50 μL volume of resiquimod or TransCon TLR7/8 Agonist (Ascendis Pharma, Inc.) was IT administered once on D0 or day 1 (D1), as indicated. Starting on D0, recombinant human IL-2 (Peprotech) was administered intraperitoneally twice daily for 5 days, followed by a 3-day dosing holiday, then administered once daily for another 5 days. Starting on D0, 60 μg of anti-PD1 (mIgG1 D265A P329G variant of clone RMP1-14, Chempartner) was administered intraperitoneally twice weekly for 2 weeks.

### Tissue collection and processing

In-life bleeds were performed by retrobulbar sinus puncture under isoflurane anesthesia or submandibular vein puncture in unanesthetized animals. Terminal blood collection was through cardiac puncture. Plasma was prepared by collecting blood in standard plasma vials containing lithium heparin on ice followed by centrifugation at 2000×*g* for 5 min at 4 °C (Charles River) or 9391×*g* for 10 min at 4 °C (Ascendis Pharma, Inc.). Plasma was transferred to new tubes and stored at −80 °C. Tumors for cytokine assessment were flash frozen in liquid nitrogen and homogenized in liquid nitrogen via mortar and pestle. Peripheral blood mononuclear cells (PBMCs) were isolated from whole blood using RBC lysis buffer (Gems Bio) following manufacturer’s recommendations. tDLNs were harvested and mechanically disrupted to obtain a single cell suspension. For tumor-infiltrating lymphocyte (TIL) isolation, harvested tumors were mechanically disrupted and digested using the mouse Tumor Dissociation Kit (Miltenyi Biotec) and gentleMACS Octo Dissociator (Miltenyi Biotec), following manufacturer’s recommendations. Cell aliquots were frozen in CryoStor (STEMCELL Technologies) following manufacturer’s recommendations.

### Cytokine quantification

Plasma cytokines/chemokines were assessed using the ProcartaPlex^™^ Multiplex Immunoassay Cytokine and Chemokine 36-Plex Mouse ProcartaPlex^™^ Panel 1A (Thermo Fisher) with results collected on a Bio-Plex 200 (Thermo Fisher) and analyzed with Bioplex Manager 6.1.1 software (Thermo Fisher), or using the Proinflammatory Panel 1 and Cytokine Panel 1 kits for mice (Meso Scale Diagnostics) with results collected on a MESO QuickPlex (Meso Scale Diagnostics) and analyzed with Discovery Workbench 4.0 (Meso Scale Diagnostics), per manufacturer’s directions. For tumor cytokines, frozen homogenized samples were lysed in 400 μl lysis buffer (Thermo Fisher) per 50 mg powder and sonicated on ice. Samples were centrifuged at 13,000*g* for 20 min at 4 °C and the supernatant was removed and snap frozen in liquid nitrogen. Protein concentrations were determined with the DC™ Protein Assay (BioRad) per manufacturer’s directions, and samples were diluted with PBS to a concentration of 5.5 mg/ml. Diluted samples were used for analyte quantitation using the ProcartaPlex^™^ Multiplex Immunoassay Cytokine and Chemokine 36-Plex Mouse ProcartaPlex^™^ Panel 1A (Thermo Fisher), per manufacturer’s directions, with results collected on a Bio-Plex 200 (Thermo Fisher) and analyzed with Bioplex Manager 6.1.1 software (Thermo Fisher). Values at or below the lower limit of quantitation (LLOQ) were recorded as one-half LLOQ for data analysis.

### Flow cytometry

For extracellular antigens, freshly isolated cells were stained with antibodies for 20 min at 4 °C in FACS buffer (PBS + 0.5% BSA), then washed and acquired by flow cytometry. For intracellular antigens, freshly isolated cells were stained for extracellular antigens, then fixed and permeabilized (FoxP3/Transcription Factor Staining Buffer Set, eBioscience), then stained for intracellular antigens for 16 h at 4 °C. For tetramer reagent staining, frozen cells were thawed and stained with tetramer or control reagent (MBL International) together with extracellular antibodies for 20 min at 4 °C in FACS buffer.

Cells were resuspended in FACS buffer before data acquisition. Cells were collected on an Agilent NovoCyte Quanteon flow cytometer, using a fixed volume to enable cell count calculations. Data was acquired using NovoExpress software (Agilent) and analyzed with FlowJo (BD Biosciences) and Ryvett (Qognit) software. Flow cytometry antibody panels and reagents used for each tissue can be found in Additional file 1: materials and methods.

### Data analysis

Data preprocessing: For FACS data, samples with CD45 count below 5000 and markers with parental cell counts below 100 were excluded from all analyses. For analyses involving multiple experiments, data were combined before analysis followed by batch-effect evaluation and correction using principal component analysis (PCA) and the removeBatchEffect function in R package limma [26].

Statistics: Repeated measures 2-way analysis of variance (ANOVA) with mixed effects was used to analyze longitudinal data from different treatment groups. Kruskal–Wallis test followed by Dunn’s post-hoc test was used for comparing different treatment groups at one or more timepoints. GraphPad Prism 9 (GraphPad Software Inc.) was used for all statistical analyses. Data were considered significant when p < 0.05.

## Results

### TransCon TLR7/8 Agonist design

**Fig. 1:**
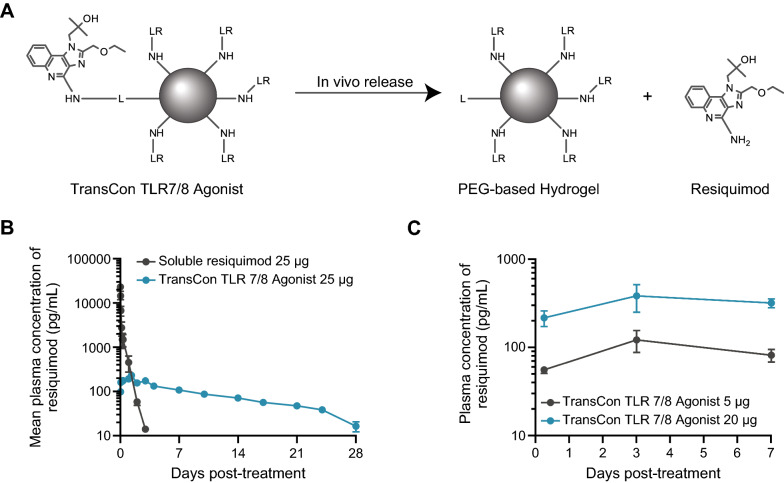
In vivo pharmacokinetic characteristics of TransCon TLR7/8 Agonist. **A** TransCon TLR7/8 Agonist was designed as a sustained-release prodrug of resiquimod consisting of resiquimod bound, at multiple conjugation sites, to a suspension of polymeric PEG hydrogel microspheres via a covalent and cleavable linker (R = resiquimod; L = linker). **B** Male Wistar rats (n = 3/group) received a single subcutaneous injection of either soluble resiquimod (25 μg) or TransCon TLR7/8 Agonist [25 μg equivalent (eq.) of resiquimod]. Blood was withdrawn and used for plasma generation over 28 days. The resiquimod concentration in the plasma samples was quantified by LC–MS/MS. Values are represented as mean resiquimod concentration (pg/ml) per group ± SEM. **C** Female BALB/c mice were SC implanted with 3 × 10^5^ CT26 tumor cells in their flank. When tumors were grown to a mean tumor volume of ~ 115 mm^3^, mice were randomized into treatment cohorts (Day 0). The day following randomization, animals received 5 or 20 μg (eq. of resiquimod) of TransCon TLR7/8 Agonist as a single intratumoral dose. Blood was withdrawn (n = 3/time point) and used for plasma generation at 6 h, 3 days and 7 days. The resiquimod concentration in the plasma samples was quantified by LC–MS/MS. Values are represented as mean resiquimod concentration (pg/ml) per dose group ± SEM

TransCon TLR7/8 Agonist was designed as a sustained-release prodrug of resiquimod, a potent TLR7/8 agonist, intended for IT administration and local retention. It consists of a suspension of polymeric polyethylene glycol (PEG) hydrogel microspheres (ca. 70–100 µm in diameter), to which resiquimod is bound through a covalent and cleavable linker at multiple conjugation sites. This TransCon linkage was designed to enable sustained resiquimod release in the TME (Fig. 1A). In vitro release of resiquimod from TransCon TLR7/8 Agonist conducted at 37 °C was analyzed by non-linear regression applying a first order fit and resulted in release half-lives of 12–14 days in the pH interval between 6.8 and 7.8 (Additional file 2: Figure S1 and manuscript in preparation).

### TransCon TLR7/8 Agonist provided slow and sustained in vivo release of resiquimod

To verify sustained release of resiquimod following local administration of TransCon TLR7/8 Agonist in vivo, a single dose of TransCon TLR7/8 Agonist or soluble resiquimod dissolved in buffer was injected subcutaneously (SC) in rats (Fig. 1B) and systemic levels of resiquimod were measured. Compared to soluble resiquimod, TransCon TLR7/8 Agonist mediated low but sustained systemic exposure of resiquimod, detectable in plasma for up to 4 weeks with a terminal half-life of around 250 h. In contrast, soluble resiquimod was cleared rapidly, with a terminal half-life of 10 h. While both treatments provided comparable AUC_Pred-∞_, the C_max_ concentrations were approximately 100-fold lower for TransCon TLR7/8 Agonist compared to soluble resiquimod (Fig. 1B). Consistently, when TransCon TLR7/8 Agonist was injected intratumorally in CT26 tumor bearing mice, a similar low but long-term and dose-proportional systemic drug exposure was observed (Fig. 1C), suggesting that the TME did not affect the release rate of resiquimod.

### TransCon TLR7/8 Agonist inhibited tumor growth and promoted local immune activation with minimal systemic cytokine induction

**Fig. 2:**
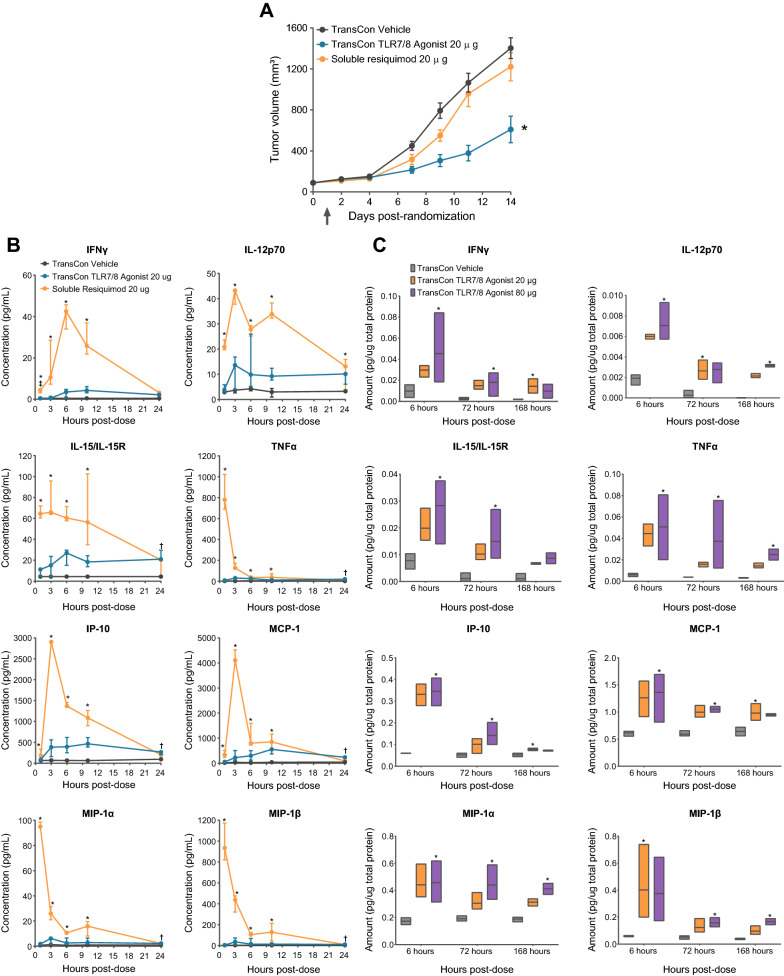
TransCon TLR7/8 Agonist promoted TGI, minimal systemic cytokine release, and sustained intratumoral cytokine expression. **A** Female BALB/c mice were SC implanted (flank) with 3 × 10^5^ CT26 tumor cells. At a mean tumor volume of ~ 90 mm^3^, mice were randomized (Day 0; n = 10–15/group). The following day, animals received either TransCon Vehicle, 20 μg (eq. of resiquimod) of TransCon TLR7/8 Agonist, or 20 μg of soluble resiquimod (single IT dose, arrow). Values are represented as mean tumor volume ± SEM. On Day 14, *p < 0.005 vs all other groups, n = 6–11/group. **B** Female BALB/c mice were SC implanted (flank) with 3 × 10^5^ CT26 tumor cells. At a mean tumor volume of ~ 120 mm^3^, mice were randomized (Day 0). The following day, animals received either TransCon Vehicle, 20 μg of TransCon TLR7/8 Agonist, or 20 μg of soluble resiquimod as a single IT dose. Plasma samples were (n = 3 independent mice/group/timepoint) assessed for cytokines by ProcartaPlex Multiplex Immunoassay. Values are represented as median plasma analyte concentration (pg/ml) with whiskers representing minimum and maximum replicate values. Points are connected for visualization purposes. *p < 0.05 Soluble Resiquimod vs TransCon Vehicle (same day), ^†^p < 0.05 TransCon TLR7/8 Agonist vs TransCon Vehicle (same day), ^‡^p < 0.05 TransCon TLR7/8 Agonist vs Soluble Resiquimod (same day). **C** Female BALB/c mice were SC implanted (flank) with 3 × 10^5^ CT26 tumor cells. At a mean tumor volume of ~ 115 mm^3^, mice were randomized (Day 0). The following day, animals received either TransCon Vehicle or 20 or 80 μg of TransCon TLR7/8 Agonist. Tumors were harvested and assessed for cytokine levels. Values are represented as the minimum, maximum, and median (black line) analyte concentrations normalized to protein input for each treatment group (n = 3/treatment/timepoint, except for the TransCon TLR7/8 Agonist treated group at the 168-h timepoint, where n = 2). *p < 0.05 vs TransCon Vehicle (same day)

TLR agonists administered intratumorally demonstrate rapid effusion from the administration site [12, 27], which may limit clinical benefit and lead to high systemic drug exposure and systemic induction of proinflammatory cytokines that promote systemic AEs such as chills, pyrexia, fatigue, nausea, and vomiting [12, 13, 27, 28]. Single IT administration of soluble resiquimod was compared to TransCon TLR7/8 Agonist containing an equivalent dose of soluble resiquimod in CT26 tumor-bearing mice and tumor growth inhibition and systemic cytokine induction were assessed. Following dosing, soluble resiquimod did not provide significant anti-tumor activity; conversely, TransCon TLR7/8 Agonist demonstrated potent and sustained anti-tumor benefit (Fig. 2A).

Systemic cytokine/chemokine analysis showed rapid and robust increases in proinflammatory cytokines and myeloid-associated chemokines following soluble resiquimod administration. However, TransCon TLR7/8 Agonist induced comparatively minimal increases in systemic cytokine/chemokine levels (Fig. 2B). Peak plasma cytokine and chemokine levels occurred between 1–6 h and between 3–10 h following soluble resiquimod and TransCon TLR7/8 Agonist dosing, respectively, with soluble resiquimod inducing up to 27-fold higher plasma cytokine/chemokine levels compared to TransCon TLR7/8 Agonist (Table 1). Despite inducing relatively low systemic cytokine and chemokine levels, a single IT administration of TransCon TLR7/8 Agonist induced robust chemokine and cytokine protein expression in the tumor (Fig. 2C). Following TransCon TLR7/8 Agonist administration, significantly elevated proinflammatory cytokine and chemokine expression was detected in the tumor over 7 days, consistent with sustained release of resiquimod from TransCon TLR7/8 Agonist within the TME (Fig. 2C).

**Table 1:** Plasma Cytokine and Chemokine C_max_/T_max_

Median C_max_ pg/ml [T_max_ h]				
Analyte	TransCon Vehicle	20 µg IT TransCon TLR7/8 Agonist	20 µg IT Soluble resiquimod	Fold difference (Soluble resiquimod C_max_/ TransCon TLR7/8 Agonist C_max_)
IFN-γ	0.465 [LLOQ]	4.28 [6]	42.5 [6]	9.9
IL-12p70	4.25 [6]	13.61 [3]	43.23 [3]	3.18
IL-15/IL-15R	4.38 [LLOQ]	26.96 [6]	65.82 [3]	2.44
TNF-α	4.64 [3]	29.83 [3]	779.94 [1]	26.15
IP-10	94.89 [24]	463.54 [10]	2904.66 [3]	6.27
MCP-1	43.61 [24]	552.49 [10]	4105.73 [3]	7.43
MIP-1α	1.31 [3]	6.13 [3]	95.02 [1]	15.5
MIP-1β	3.4 [6]	35.21 [3]	933.93 [1]	26.52

Median maximum concentration (C_max_), the time at which the median maximum concentration was measured [T_max_ in hours (h), in parentheses], and fold difference in median C_max_ between TransCon TLR7/8 Agonist and soluble resiquimod treatment for plasma analytes measured from animals described in Fig. 2B are shown

### Intratumoral TransCon TLR7/8 Agonist treatment induced low peripheral cytokine expression while promoting activation of peripheral T and B cells

**Fig. 3:**
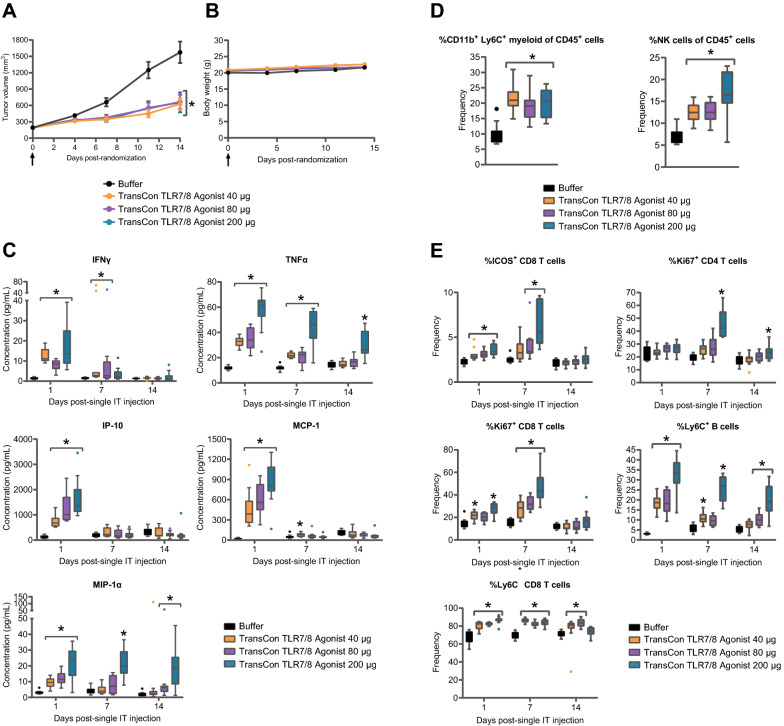
TransCon TLR7/8 Agonist associated with low systemic cytokines and sustained activation of peripheral immune cells. In two independent experiments, Female BALB/c mice were SC implanted (flank) with 2 × 10^6^ CT26 tumor cells. At a mean tumor volume of ~ 185–200 mm^3^, mice were randomized into treatment cohorts (Day 0; n = 18–20/group). On the same day of randomization, animals received either buffer control or TransCon TLR7/8 Agonist as a single IT dose (arrow). For each experiment, samples from 5 to 6 animals were collected for processing and analysis at each timepoint. Data from the two experiments was concatenated prior to analysis. **A** Mean tumor volume (mm^3^) ± SEM over time is shown (n = 13–38/group/timepoint). On Day 14, *p < 0.05 vs Buffer, n = 13–16/group. **B** Mean body weight (g) ± SEM is shown (n = 13–38/group/timepoint). **C** Plasma cytokine/chemokine concentrations over time are shown. **D** Frequency of indicated immune cell sub-type within the CD45^+^ gate assessed in PBMCs one day after treatment is shown. **E** PBMCs were isolated at various times following treatment and assessed for markers of adaptive immune-cell subsets and activation via flow cytometry. For **C**–**F** *p < 0.05 vs Buffer on the same day, n = 5–11/ group/timepoint

To determine if evidence immune activation following TransCon TLR7/8 Agonist treatment was detectable in peripheral circulation, we administered TransCon TLR7/8 Agonist (40, 80, or 200 μg resiquimod equivalent) IT in CT26 tumor bearing mice and levels of peripheral proinflammatory cytokines/chemokines and phenotypes of immune cells were evaluated. Tumor growth was significantly inhibited at all dose levels (Fig. 3A), consistent with previous dose titration experiments showing TransCon TLR7/8 Agonist inhibited CT26 (Additional file 3: Figure S2) and MC38 (Additional file 4: Figure S3) tumor growth in a dose-dependent manner. The anti-tumor effect was attributed to local resiquimod release and not a foreign body response [29], as IT administration of a hydrogel without resiquimod (TransCon Vehicle, Additional file 3: Figure S2) showed similar lack of anti-tumor activity as IT administration of buffer (Fig. 3A). The TransCon TLR7/8 Agonist was well tolerated as a monotherapy with no treatment-related body weight loss (Fig. 3B) or change in body condition score (data not shown). Furthermore, TransCon TLR7/8 Agonist was systemically well tolerated in a comprehensive safety evaluation performed in repeat-dose toxicity studies in mice (IT administration) and cynomolgus monkeys (SC administration) (data not shown). Similar to previous data (Fig. 1B), TransCon TLR7/8 Agonist led to low but detectable increases in the concentration of plasma cytokines/chemokines within 24 h of IT dosing (Fig. 3C). Most measured cytokines/chemokines displayed dose dependent induction. Notably, even at the highest dose level of 200 μg, TransCon TLR7/8 Agonist induced mean peak plasma cytokine levels that were below those induced by a much lower, 20 μg, dose of IT soluble resiquimod (Fig. 2B). IFNγ, IP-10, and MCP-1 displayed an early expression pattern with plasma levels peaking 1 day following dosing and at levels close to those of control conditions by day 7 post-dose. On the other hand, TNFα and MIP-1α displayed expression over 2 weeks, supporting pharmacodynamic activity consistent with the sustained drug release observed in vivo (Fig. 1B, C).

Pharmacodynamic changes in peripheral immune subsets following IT TransCon TLR7/8 Agonist treatment using flow cytometry was next assessed. An early increase in the frequency of CD11b^+^ Ly6C^+^ monocytic myeloid cells was detected in CD45^+^ peripheral blood mononuclear cells (PBMCs) 24 h after TransCon TLR7/8 Agonist treatment (Fig. 3D). This result is consistent with the increase in MCP-1 (Fig. 3C)—an important chemokine for egress of monocytes from the bone marrow [30] that can promote recruitment of proinflammatory Ly6C^+^ monocytic myeloid cells to sites of inflammation [31]. Similarly, following TransCon TLR7/8 Agonist treatment, an early increase in NK cell frequency in PBMCs was also observed (Fig. 3D), correlating with an early increase in plasma IP-10 concentrations (Fig. 3C). Evidence of dose-dependent activation of peripheral adaptive immune cells was also observed. Peripheral CD8^+^ T cells displayed an increased frequency of cells positive for the activation and costimulatory marker ICOS, detectable 1- and 7-days following treatment. An increased frequency of cells expressing the Ki67 proliferation marker was detectable in both CD8^+^ and CD4^+^ T cell subsets up to day 7 and 14, respectively, after TransCon TLR7/8 Agonist treatment. Finally, an increased frequency of cells expressing the memory marker, Ly6C, was also observed on both CD8^+^ T cell and B cell populations throughout the study (Fig. 3E).

### Intratumoral TransCon TLR7/8 Agonist potentiated activation of antigen-presenting cells in tDLNs and tumors while promoting activation of adaptive and cytotoxic immune cells

**Fig. 4:**
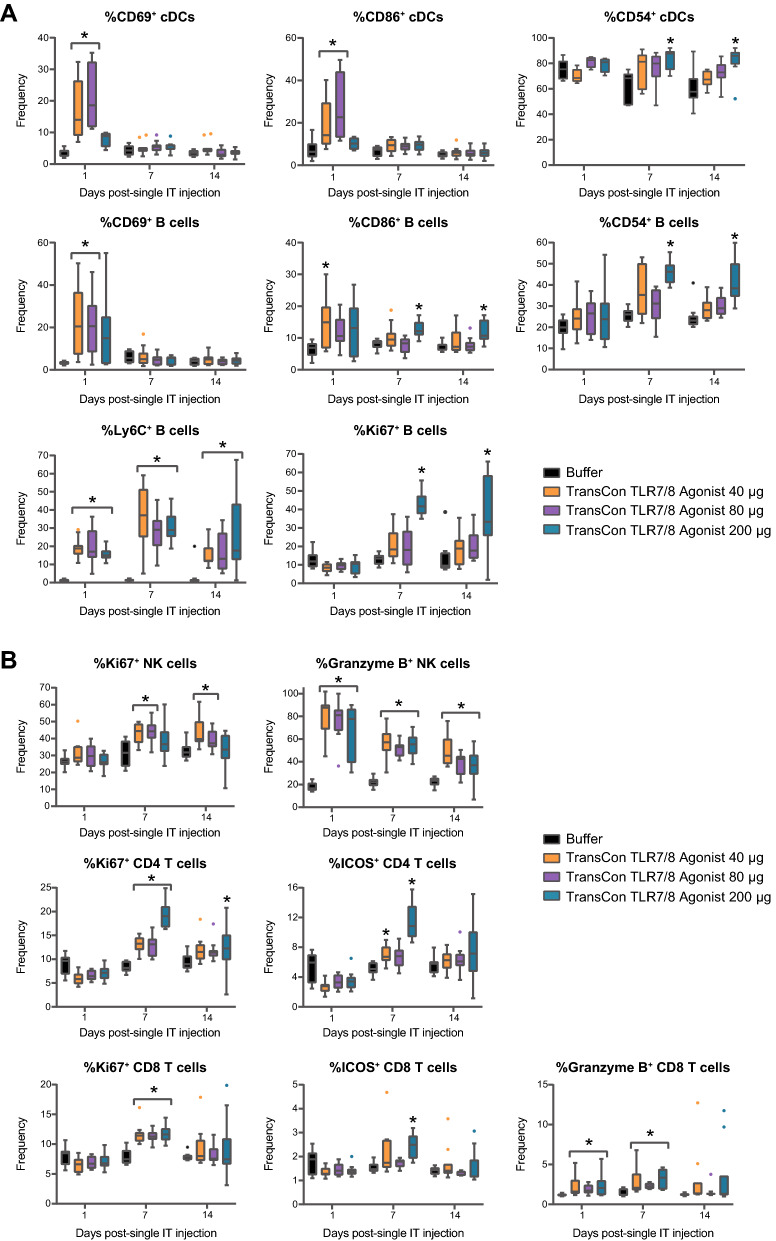
IT TransCon TLR7/8 Agonist potentiated activation of APCs and T cells in tDLNs. Mice were treated as described in Fig. 3. Lymphocytes from tDLNs were isolated at various times following treatment and assessed for markers of immune-cell subsets and activation via flow cytometry. **A** Antigen presenting cell immunophenotyping. **B** Adaptive and cytolytic immune cell immunophenotyping. For **A** and **B** *p < 0.05 vs Buffer on the same day, n = 9–11/group/timepoint

Activation of APCs, such as conventional dendritic cells (cDCs), macrophages, and B cells, via TLR agonism can enhance tumor antigen uptake and presentation of said antigens on MHC to helper CD4^+^ and cytotoxic CD8^+^ T cells [32] as well as recruit and activate innate cytotoxic lymphocytes such as NK cells. Tumor antigen uptake and presentation to T cells in tumor-draining lymph nodes (tDLN) and/or tumor-associated lymphoid tissue is critical for the development of efficient anti-tumor immune responses [33]. To understand whether IT administered TransCon TLR7/8 Agonist could promote APC, cytotoxic, and helper T cell activation, the expression of markers of activation and effector phenotype in innate and adaptive immune cell subsets in tDLN following TransCon TLR7/8 Agonist treatment was evaluated. Following IT administration of a single dose of TransCon TLR7/8 Agonist, we observed early upregulation of the activation marker CD69 and costimulatory molecule CD86 on cDCs and B cells in the tDLN (Fig. 4A). A dose-dependent upregulation of the activation marker CD54 [34], was observed on both cDCs and B cells by 7 days post-treatment and was detectable at day 14 in both cell subsets. Further, B cells displayed induction of Ki67 at 7- and 14-days post-treatment and significantly upregulated Ly6C, a marker induced on activated and plasma-like B cells [35], at all timepoints following TransCon TLR7/8 Agonist treatment (Fig. 4A). Evidence of activation of NK and T cell subsets was also observed in tDLNs (Fig. 4B). Ki67 was elevated on NK cells and CD4^+^ T cells on days 7 and 14, and on CD8^+^ T cells on day 7 post-treatment. Furthermore, ICOS upregulation was detected at 7 days post-dose on both CD4^+^ and CD8^+^ T cells. Enhanced expression of granzyme B in both cytotoxic NK and CD8^+^ T cells was also observed (Fig. 4B).

**Fig. 5:**
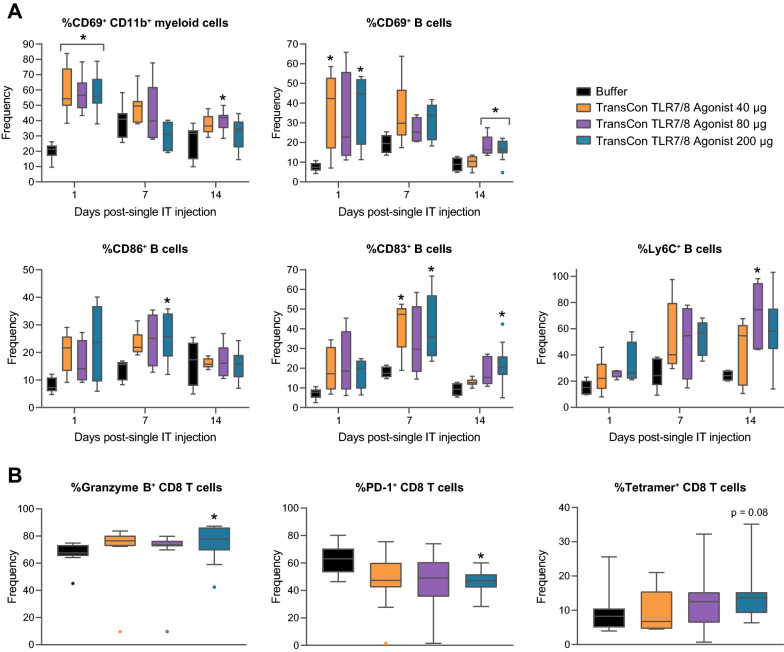
IT TransCon TLR7/8 Agonist potentiated activation of APCs and antigen-specific CD8 T cells in tumors. Mice were treated as described in Fig. 3. Dissociated tumor cells were isolated at various times following treatment and assessed for markers of immune-cell subsets and activation via flow cytometry. **A** Antigen presenting cell immunophenotyping following treatment initiation. *p < 0.05 vs Buffer on the same day, n = 5–11 per group per timepoint. **B** CD8 T cell granzyme B and PD-1 immunophenotyping on day 14 following treatment initiation. *p < 0.05 vs Buffer, n = 9–11/group. **C** %AH1-tetramer^+^ CD8 T cells on Day 14 following treatment initiation. n = 9–11/group

To assess whether APCs showed evidence of activation in the TME following TransCon TLR7/8 Agonist treatment, we evaluated activation marker expression on APC subsets from tumor samples at various times post-treatment. Consistent with the tDLN, TME APCs demonstrated evidence of increased CD69 on CD11b^+^ myeloid cells and B cells following a single IT administration of TransCon TLR7/8 Agonist (Fig. 5A). Costimulatory markers were also upregulated on B cells following TransCon TLR7/8 Agonist treatment, with CD86 displaying upregulation 7 days after treatment, and CD83 displaying upregulated expression 7 and 14 days after treatment. Similar to tDLN B cells, TME B cells displayed elevated expression of activation marker Ly6C following IT TransCon TLR7/8 Agonist treatment (Fig. 5A).

We assessed tumor-infiltrating CD8^+^ T cells for expression of the effector lytic molecule, granzyme B, as well as the inhibitory PD-1 receptor. 14 days post-dose, a significant increase in the percentage of granzyme B positive CD8^+^ T cells was observed, with a concomitant decrease in the percentage of PD-1 expressing CD8^+^ T cells (Fig. 5B). We further evaluated the frequency of tumor epitope-specific CD8^+^ T cells using H-2L^d^ tetramers loaded with the murine leukemia virus (MuLV) GP70 AH1 peptide as CT26 cells endogenously express this antigen [36]. In accordance with the described upregulation of APC function, a trend (p = 0.08) of increased frequencies of AH1-tetramer^+^ CD8^+^ T cells was observed 14 days following TransCon TLR7/8 Agonist treatment compared to the Buffer group (Fig. 5C).

### Intratumoral TransCon TLR7/8 Agonist combines with systemic immunotherapy to promote anti-tumor activity and immunological memory

**Fig. 6:**
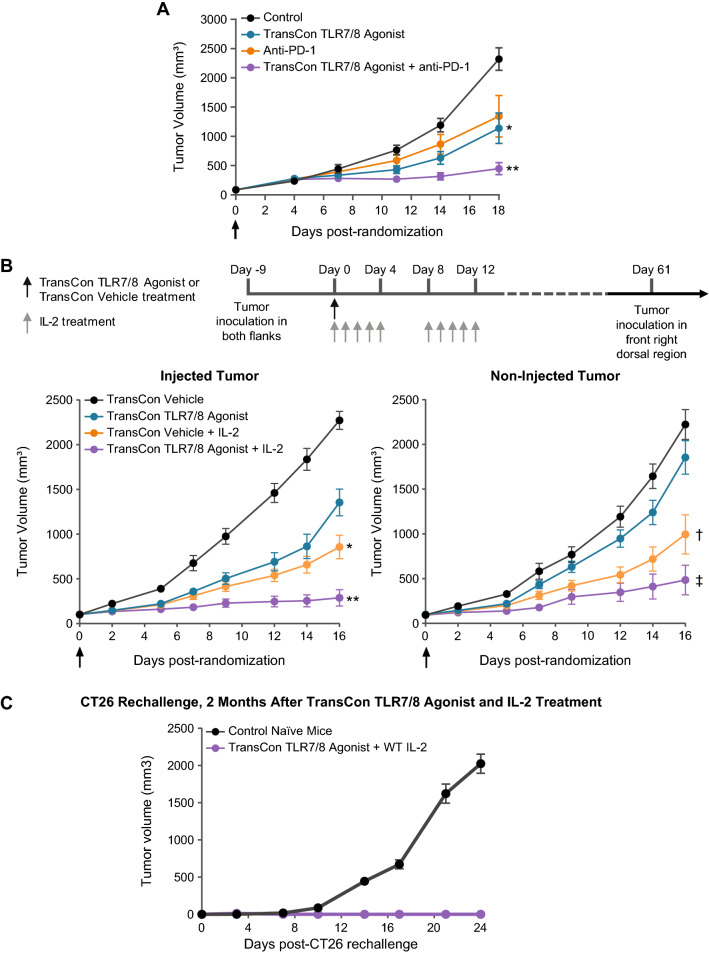
TransCon TLR7/8 Agonist with immunotherapy promoted TGI in injected and non-injected tumors and immunological memory. **A** Female BALB/c mice were SC implanted (flank) with 2 × 10^6^ CT26 tumor cells. At a mean tumor volume of ~ 80 mm^3^, mice were randomized into treatment cohorts (Day 0; n = 10/group). On the same day of randomization, animals received either anti-PD1, 100 μg (eq. of resiquimod) TransCon TLR7/8 Agonist as a single IT dose (arrow) or were left untreated (Control). On Day 18, *p < 0.05 vs Control, and **p < 0.05 vs control, n = 9–10/group. **B** Female BALB/c mice were SC implanted with 5 × 10^5^ CT26 tumor cells into the left and right flanks. At a mean tumor volume of ~ 100 mm^3^, mice were randomized into treatment cohorts (Day 0). On the day of randomization, animals received either TransCon Vehicle or 216 μg (eq. of resiquimod) of TransCon TLR7/8 Agonist as a single intratumoral dose (arrow). Some cohorts were further treated with 20 μg human IL-2 dosed twice daily on Days 0–4 and once daily on Days 8–12. In this experiment, 3 out of 7 mice treated with TransCon TLR7/8 Agonist + IL-2 experienced complete regressions in injected and non-injected tumors. On Day 16, *p < 0.05 vs TransCon Vehicle and **p < 0.05 vs all other groups, n = 7/group. For non-injected tumors on Day 18, ^†^p < 0.05 vs Control and ^‡^p < 0.05 vs Control and TransCon TLR7/8 Agonist, n = 7/group. **C** Mice from **B** that were treated with TransCon TLR7/8 Agonist and IL-2, and that experienced complete regressions in both treated and untreated tumors for at least 10 days (n = 3), were SC rechallenged with CT26 tumor cells in their front right dorsal region and observed for tumor growth. Naïve mice (n = 10) were used as controls for tumor growth. Values are represented as mean tumor volume ± SEM

Given the observation that TransCon TLR7/8 Agonist monotherapy induced activation of both innate (NK) and adaptive (CD8^+^) cytolytic cell types, we hypothesized that it could also potentiate anti-tumor efficacy when combined with systemic immunotherapy. We treated CT26 tumor bearing mice with a single IT dose of TransCon TLR7/8 Agonist either with or without systemic anti-PD1 (Fig. 6A). As monotherapy, TransCon TLR7/8 Agonist mediated tumor growth inhibition. Importantly, combination treatment with TransCon TLR7/8 Agonist and anti-PD1 antibody yielded greater anti-tumor activity over either agent alone, suggesting that T cell promoting immunotherapies may further potentiate anti-tumor responses in TransCon TLR7/8 Agonist treated tumors. Consistent with this hypothesis, a single dose of TransCon TLR7/8 Agonist in combination with systemic recombinant IL-2 resulted in greater anti-tumor activity in injected and non-injected tumors in an abscopal model, with mice bearing CT26 tumors on both flanks, compared to either single agent treatment alone (Fig. 6B). Within the combination group, 3 out of 7 mice experienced complete regressions of both tumors. Upon tumor rechallenge with CT26 cells, none of these previously treated mice displayed tumor growth (Fig. 6C), demonstrating that the combination of TransCon TLR7/8 Agonist with systemic immunotherapy promoted the functional generation of a robust anti-tumor immune memory response.

## Discussion

Based on their ability to stimulate both innate and adaptive immunity, TLR agonists are promising cancer immunotherapy agents. However, systemic administration of TLR agonists typically demonstrate unfavorable pharmacokinetic properties (short half-life), a narrow therapeutic index, and toxicity as evident by systemic cytokine release [37–39]. Newer approaches have used local IT administration of TLR agonists, however these still often lead to rapid systemic drug exposure, resulting in systemic proinflammatory cytokine release and emergence of AEs in patients [16, 40, 41], possibly limiting therapeutic potential. Frequent IT dosing of TLR agonists is often used to help mitigate some of the described shortcomings, but this is not practical in a clinical setting. Several strategies to promote retention of TLR agonists in the TME to facilitate longer drug exposure have been or are being evaluated both preclinically and clinically. One example is a soluble prodrug containing resiquimod conjugated to a four-arm PEG backbone. Preclinical data supported improved anti-tumor efficacy of this prodrug compared to soluble resiquimod, however as a soluble prodrug it was absorbed into systemic circulation and was detected in the periphery within hours in both preclinical models [42] and in patients [12]. Thus, release of active resiquimod in the TME may be dictated by the absorption kinetics of the soluble prodrug. Other carrier-based technologies using resiquimod as a payload, including surgically implantable hydrogels [43] and various nanoparticle-based carriers [44–48], have also been preclinically characterized; however, additional translational studies in patients are needed to demonstrate the practicality of the delivery method and the desired tumor retention, respectively. To address these issues, TransCon TLR7/8 Agonist was designed to provide sustained IT delivery of resiquimod while minimizing systemic exposure and related AEs.

In the present study, we describe the preclinical evaluation of TransCon TLR7/8 Agonist as an IT administered therapy for treating cancer. The data supported sustained release of resiquimod for weeks following administration of a single dose of TransCon TLR7/8 Agonist in rats (SC) and mice (IT). When compared to a locally administered equimolar dose of soluble resiquimod, TransCon TLR7/8 Agonist displayed far lower systemic C_max_ of drug, suggesting potential for lower cytokine release associated with systemic exposure to active drug. Sustained local resiquimod release in the TME is predicted to facilitate durable anti-tumor immunity with minimal systemic cytokine release. Indeed, a single IT administration of TransCon TLR7/8 Agonist was sufficient to induce robust and persistent anti-tumor activity, while also inducing significantly lower systemic cytokine/chemokine release compared to administration of soluble resiquimod. These data support the hypothesis that TransCon TLR7/8 Agonist may enable sustained resiquimod exposure in the TME, promoting more efficient anti-tumor activity compared to soluble resiquimod treatment, and may limit peripheral exposure of resiquimod, potentially mitigating systemic adverse events. Pockros et al. [38] demonstrated that in patients with chronic hepatitis C infection, oral administration of resiquimod led to rapid systemic drug exposure which positively correlated with the induction of systemically high proinflammatory cytokine levels and severe AEs in a dose-dependent manner. At a dose level of 0.02 mg/kg, which corresponded to a plasma resiquimod C_max_ of 7.55 ± 4.17 ng/ml, 73% of patients experienced severe AEs that correlated with cytokine release [38]. However, data from resiquimod exposure following IT administration of TransCon TLR7/8 Agonist in rodents, and SC administration in cynomolgus monkeys (data not shown), showed resiquimod exposure well below the C_max_ reported to elicit severe AEs in humans [38] despite higher equivalent resiquimod dose levels per kilogram. Earlier preclinical studies suggested that repeated systemic resiquimod exposure may lead to TLR tolerance and impaired anti-tumor responses as typified by significantly reduced systemic cytokine release and hyporesponsiveness in TLR7-expressing myeloid cells [49]. However, our data demonstrated elevated cytokine expression in the TME over 7 days following TransCon TLR7/8 Agonist administration, mirroring drug exposure data, suggesting that resiquimod released from TransCon TLR7/8 Agonist promoted a local proinflammatory innate immune response in the TME.

Accordingly, studies in CT26 tumor-bearing mice indicated that IT TransCon TLR7/8 Agonist treatment mediated potent single agent anti-tumor activity in a dose-dependent manner, with a favorable tolerability profile at all dose levels tested, as indicated by lack of treatment-related weight loss or change in body condition score in treated animals. The good tolerability across all doses tested is likely due to the low systemic cytokine/chemokine expression induced by TransCon TLR7/8 Agonist treatment compared to soluble resiquimod treatment. Despite low induction of cytokine/chemokine levels in the periphery, evidence of on-target pharmacodynamic activity was observed based on elevated levels of systemic proinflammatory cytokines/chemokines for at least 2 weeks post-dose. It is not clear whether the observed and differential kinetics of cytokine/chemokine induction following TransCon TLR7/8 Agonist are due to direct TLR7/8 agonism (e.g. TNFα and MIP-1α) or may be, in part, due to secondary induction downstream of cytokine receptor activation (e.g. IFNγ, MCP-1, and IP-10). Regardless, early systemic chemokine induction following TransCon TLR7/8 Agonist treatment correlated with an increase in peripheral NK and myeloid cells, suggesting mobilization of cells that may participate in tumor killing and tumor antigen presentation, respectively. MCP-1 induction associated with TransCon TLR7/8 Agonist treatment may promote myeloid cell egress from bone marrow and may promote myeloid cell trafficking to treated tumors. Furthermore, IP-10, which can be induced with resiquimod treatment [50] or in response to IFNγ [51] and is an important TH1-associated mediator of NK and adaptive immune cell recruitment [52], may participate in adaptive and cytotoxic immune cell recruitment to the TME. Consistently, we saw evidence of activated NK cells in tDLNs as well as activated APCs in both tDLNs and tumors, following TransCon TLR7/8 Agonist treatment.

B cells also showed evidence of robust activation in peripheral blood, tDLN, and tumor with treatment. Furthermore, we observed evidence of treatment-related plasma-like cell generation, as indicated by expression of Ly6C on B cells [35] in all tissue compartments assessed. B cells and B cell-derived plasma-like cells have potent antigen presenting cell functions in both tumor and tumor-draining lymph nodes and can secrete anti-tumor antibodies which may facilitate tumor killing [53]. Given the observed activation of B cells following TransCon TLR7/8 Agonist treatment, an enhanced production of anti-tumor antibodies may contribute to anti-tumor activity and warrants further investigation. Furthermore, tumor-infiltrating plasma-like cells have been linked to the formation of tertiary lymphoid structures—inducible lymphoid aggregates—associated with better patient outcomes, where intratumoral antigen presentation can take place [54, 55]. Tumor-antigen uptake and presentation in the TME and tumor-associated lymphoid tissues promote systemic tumor-antigen specific immunity [33]. Additionally, TLR agonism can promote upregulation of costimulatory cell-surface molecules that can further activate cytotoxic and helper T cells [4]. Indeed, IT delivery of TransCon TLR7/8 Agonist led to an increase in activated CD8 and CD4 T cells in peripheral blood and tDLN, which may contribute to systemic anti-tumor responses.

Importantly, TransCon TLR7/8 Agonist therapy led to an increased frequency of granzyme-positive, and lower frequency of PD-1 positive, tumor-infiltrating CD8 T cells. Previous reports suggest TLR activation may lower PD-1 expression during T cell stimulation and promote more robust tumor antigen-specific effector responses [56, 57]. Consistent with this hypothesis, we observed an increase in the frequency of tumor-antigen-specific CD8 T cells with TransCon TLR7/8 Agonist. The data presented suggest TransCon TLR7/8 Agonist primarily promotes innate immune and antigen presenting cell activation, which may be potentiated by combination therapy with agents stimulating the adaptive immune system, such as T cell targeted immunotherapies. Accordingly, anti-PD1 and IL-2 potentiated anti-tumor responses in TransCon TLR7/8 Agonist treated tumors. Combination studies with IL-2 also included assessment of non-injected tumors and the results illustrated efficacy in both injected and non-injected tumors, including complete responses. These data suggest that TransCon TLR7/8 Agonist treatment may promote tumor antigen presentation and priming of tumor-specific cytotoxic T cells associated with treated tumors, while IL-2 may promote further expansion of said tumor-specific CD8 T cells, allowing them to exert systemic tumor killing activity, resulting in regression of tumors not treated with TransCon TLR7/8 Agonist. Moreover, mice that showed complete responses following treatment with TransCon TLR7/8 Agonist and IL-2 did not develop palpable tumors following tumor rechallenge, indicating immunological memory developed with the combination treatment during the initial tumor challenge.

## Conclusions

Taken together, the described studies provide proof of principle that a single dose of TransCon TLR7/8 Agonist mediates anti-tumor efficacy via sustained delivery of resiquimod to the tumor site while avoiding high systemic exposure and reducing the risk of AEs. These studies also demonstrate that a single dose of TransCon TLR7/8 Agonist may promote systemic adaptive anti-tumor immunity and immune memory and may be combined with complementary immunotherapies for increased efficacy in patients. These data suggest that sustained resiquimod exposure in the TME, mediated by TransCon TLR7/8 Agonist, has the potential to provide clinical benefit to patients and dramatically improve the practicality of intratumoral therapies by enabling infrequent dosing. The data presented suggest TransCon TLR7/8 Agonist may promote both innate and adaptive immune cell-driven anti-tumor activity. Immunologically “cold” tumors, such as pancreatic and prostate [58], may benefit from TransCon TLR7/8 Agonist therapy via innate immune cell activation, promotion of tumor antigen presentation, and recruitment of tumor-specific cytolytic T cells to the TME. On the other hand, immunologically “hot” tumors such as melanoma, head and neck, and lung [58], that are generally more sensitive to immune checkpoint blockade may also benefit from TransCon TLR7/8 Agonist through further potentiation of antigen presentation and of induction of local inflammation. Potentiation of antigen presentation via TransCon TLR7/8 Agonist therapy in both “cold” and hot” tumors could further benefit from the additional combinations with T cell activating immunotherapies such as IL-2, including IL-2 variants, or anti-PD1. Furthermore, compared to unmodified TLR agonists, the sustained release nature of TransCon TLR7/8 Agonist may allow for greater patient access by facilitating less frequent treatment visits thus making intratumoral delivery more practical not only for more accessible tumors (e.g., melanoma and head and neck) but also for deeper tumors (e.g., lung). A clinical trial to evaluate the safety and efficacy of TransCon TLR7/8 Agonist, as monotherapy and in combination with pembrolizumab, in cancer patients is currently ongoing (transcendIT-101; NCT04799054).

## Supplementary Information

**Additional file 1: Table S1A.** Flow cytometry reagents for analysis of mouse blood. **Table S1B.** Populations analyzed in mouse blood. **Table S2A.** Flow cytometry reagents for analysis of antigen presenting cells from mouse tDLN and tumor cell samples. **Table S2B.** Antigen presenting cell populations analyzed in mouse tDLN and tumor cell samples. **Table S3A.** Flow cytometry reagents for analysis of lymphoid cells from mouse tDLN and tumor cell samples. **Table S3B.** Lymphoid cell populations analyzed in mouse tDLN and tumor cell samples. **Table S4A.** Flow cytometry reagents for analysis of AH1-tetramer^+^ cells from mouse tumor cell samples. **Table S4B.** AH1-tetramer^+^ cell populations analyzed in mouse tumor samples

**Additional file 2: Figure S1.** Consistent resiquimod release in vitro from TransCon TLR7/8 Agonist in the pH range between 6.8 and 7.8. A suspension of TransCon TLR7/8 Agonist (nominal 47 μg eq. of resiquimod) in 60 mM phosphate buffer was incubated at 37 °C at various pH levels. Samples of the supernatant were withdrawn at various times and the resiquimod content was determined. Values are represented as mean percentage from 2 experiments of resiquimod release of total resiquimod loaded on the hydrogel. Release half-lives were determined following a first-order fit of the data.

**Additional file 3: Figure S2.** TransCon TLR7/8 Agonist inhibited CT26 tumor growth in a dose-dependent manner. Female BALB/c mice were SC implanted with 3 x 105 CT26 tumor cells in their flank. When tumors were grown to a mean tumor volume of ~115 mm3, mice were randomized into treatment cohorts (Day 0; n = 17/group). The day following randomization, animals received either empty hydrogel (TransCon Vehicle) or 5, 20, 80, or 200 μg (eq. of resiquimod) of TransCon TLR7/8 Agonist as a single intratumoral dose (arrow). Values are represented as mean tumor volume ± SEM. On Day 10, * = p<0.05 vs TransCon Vehicle, n = 7-8/group.

**Additional file 4: Figure S3.** TransCon TLR7/8 Agonist inhibited MC38 tumor growth in a dose-dependent manner. Female C57BL/6 mice were SC implanted with 5 x 105 CT26 tumor cells in their flank. When tumors were grown to a mean tumor volume of ~100 mm3, mice were randomized into treatment cohorts (Day 0; n = 20-22/group). On the same day of randomization, animals received either empty hydrogel (TransCon Vehicle) or 25 or 134 μg (eq. of resiquimod) of TransCon TLR7/8 Agonist as a single intratumoral dose (arrow). Values are represented as mean tumor volume ± SEM. On Day 18, * = p<0.05 vs TransCon Vehicle, n = 13-17/group.

## Data Availability

The datasets used and/or analyzed during the current study are available from the corresponding author on reasonable request.

## Abbreviations

AE: Adverse event
ANOVA: Analysis of variance
APC: Antigen presenting cell
cDC: Conventional dendritic cells
C_max_: Maximum concentration
CTCL: Cutaneous T cell lymphoma
DMEM: Dulbecco's modified eagle medium
eq.: Equivalent
IT: Intratumoral
LC–MS/MS: Liquid chromatography with tandem mass spectrometry
LLOQ: Lower limit of quantitation
MuLV: Murine leukemia virus
NK: Natural killer
PBMC: Peripheral blood mononuclear cells
PBS: Phosphate buffered saline
PCA: Principal component analysis
PEG: Polyethylene glycol
tDLN: Tumor-draining lymph node
TIL: Tumor-infiltrating lymphocytes
TLR: Toll-like receptor
T_max_: Time to reach C_max_
TME: Tumor microenvironment
TransCon: Transient conjugation
SC: Subcutaneously
