# Supplementary material for: Intratumoral delivery of TransCon™ TLR7/8 Agonist promotes sustained anti-tumor activity and local immune cell activation while minimizing systemic cytokine induction
Source: Cancer Cell Int. 2022 Sep 19;22:286. doi: 10.1186/s12935-022-02708-6 (PMC9484246; doi:10.1186/s12935-022-02708-6)
Supplement: Supplementary file 1 — Additional file 1: Table S1A. Flow cytometry reagents for analysis of mouse blood. Table S1B. Populations analyzed in mouse blood. Table S2A. Flow cytometry reagents for analysis of antigen presenting cells from mouse tDLN and tumor cell samples. Table S2B. Antigen presenting cell populations analyzed in mouse tDLN and tumor cell samples. Table S3A. Flow cytometry reagents for analysis of lymphoid cells from mouse tDLN and tumor cell samples. Table S3B. Lymphoid cell populations analyzed in mouse tDLN and tumor cell samples. Table S4A. Flow cytometry reagents for analysis of AH1-tetramer+ cells from mouse tumor cell samples. Table S4B. AH1-tetramer+ cell populations analyzed in mouse tumor samples [file 12935_2022_2708_MOESM1_ESM.docx]

**Additional Methods**

**Flow cytometry reagents and cell subset gating definitions**

The following antibodies and gating definitions were used for blood immunophenotyping. Frequencies for each cell subset within their respective parental gate were determined.

Table S1 A: Flow cytometry reagents for analysis of mouse blood

| Marker / Reagent | Fluorochrome | Cat. | Clone | Vendor |
| --- | --- | --- | --- | --- |
| CD25 | APC | 17-0251-82 | PC61.5 | eBioscience |
| CD45 | APC-eF780 | 47-0451-82 | 30-F11 | eBioscience |
| CD4 | BV650 | 100469 | GK1.5 | BioLegend |
| CD19 | PE-Cy5 | 115509 | 6D5 | BioLegend |
| I-A/I-E (MHC II) | BV421 | 107632 | M5/114.15.2 | BioLegend |
| Ly6C | AF488 | 53-5932-82 | HK1.4 | eBioscience |
| CD3e | PerCP-eF710 | 46-0033-82 | eBio500A2 | eBioscience |
| CD8a | AF700 | 56-0081-82 | 53-6.7 | eBioscience |
| CD11b | BV785 | 101243 | M1/70 | BioLegend |
| CD69 | BV711 | 104537 | H1.2F3 | BioLegend |
| ICOS | BV605 | 313537 | C398.4A | BioLegend |
| NKp46 (CD335) | PE-eF610 | 61-3351-82 | 29A1.4 | eBioscience |
| Ly6G | BV570 | 127629 | 1A8 | BioLegend |
| FoxP3 | PE | 12-5773-80 | FJK-16s | eBioscience |
| Ki67 | PE-Cy7 | 561283 | B56 | BD |
| Fc Block (anti-CD16/CD32) | Purified | 14-0161-86 | 93 | eBioscience |
| Live/Dead | eF506 | 65-0866-14 | Not applicable | eBioscience |

Table S1 B: Populations analyzed in mouse blood

| Population | Cell Subset Gating Definition |
| --- | --- |
| CD11b^+^ Ly6C^+^ myeloid cells of CD45^+^ cells | Singlets>Live cells>CD45^+^CD19^-^CD3^-^CD11b^+^Ly6C^+^ |
| NK cells of CD45^+^ cells | Singlets>Live cells> CD45^+^CD19^-^CD3^-^NKp46^+^ |
| CD8^+^ T cells | Singlets>Live cells>CD45^+^CD19^-^CD3^+^CD8^+^ |
| ICOS^+^ CD8^+^ T cells | Singlets>Live cells>CD45^+^CD19^-^CD3^+^CD8^+^ICOS^+^ |
| KI67^+^ CD8^+^ T cells | Singlets>Live cells>CD45^+^CD19^-^CD3^+^CD8^+^Ki67^+^ |
| Ly6C^+^ CD8^+^ T cells | Singlets>Live cells>CD45^+^CD19^-^CD3^+^CD8^+^Ly6C^+^ |
| CD4^+^ T cells | Singlets>Live cells>CD45^+^CD19^-^CD3^+^CD4^+^ |
| KI67^+^ CD4^+^ T cells | Singlets>Live cells>CD45^+^CD19^-^CD3^+^CD4^+^Ki67^+^ |
| B cells | Singlets>Live cells>CD45^+^CD19^+^ |
| Ly6C^+^ B cells | Singlets>Live cells>CD45^+^CD19^+^Ly6C^+^ |

The following antibodies and gating definitions were used for antigen presenting cell immunophenotyping for tDLNs and tumor cell samples. Frequencies for each cell subset within their respective parental gate were determined.

Table S2 A: Flow cytometry reagents for analysis of antigen presenting cells from mouse tDLN and tumor cell samples

| Marker / Reagent | Fluorochrome | Cat. | Clone | Vendor |
| --- | --- | --- | --- | --- |
| I-A/I-E (MHC II) | FITC | 107606 | M5/114.15.2 | BioLegend |
| CD19 | PE-Cy5 | 115509 | 6D5 | BioLegend |
| Ly6C | APC | 128016 | HK1.4 | BioLegend |
| CD3e | PerCP-eF710 | 46-0033-82 | eBio500A2 | eBioscience |
| CD54 | APC/Fire750 | 116126 | YN1/1.7.4 | BioLegend |
| CD11c | BV605 | 117334 | N418 | BioLegend |
| PD-L1 (CD274) | BV650 | 124336 | 10F.9G2 | BioLegend |
| CD69 | BV711 | 104537 | H1.2F3 | BioLegend |
| CD11b | BV785 | 101243 | M1/70 | BioLegend |
| F4/80 | PE | 123110 | BM8 | BioLegend |
| Ly6G | PE-eFluor 610 | 61-9668-82 | 1A8-Ly6G | eBioscience |
| CD83 | PE-Cy7 | 121517 | Michel-19 | BioLegend |
| CD206 (MMR) | BV421 | 141717 | C068C2 | BioLegend |
| CD45 | BV570 | 103135 | 30-F11 | BioLegend |
| NKp46 (CD335) | PerCP-eFluor710 | 46-3351-82 | 29A1.4 | eBioscience |
| CD86 | AF700 | 105024 | GL-1 | BioLegend |
| Fc Block (anti-CD16/CD32) | Purified | 14-0161-86 | 93 | eBioscience |
| Live/Dead | eF506 | 65-0866-14 | Not applicable | eBioscience |

Table S2 B: Antigen presenting cell populations analyzed in mouse tDLN and tumor cell samples

| Population | Cell Subset Gating Definition |
| --- | --- |
| cDCs | Live cells>CD45^+^ Singlets> CD19^-^CD11b^-^CD11c^+^ |
| CD69^+^ cDCs | Live cells>CD45^+^ Singlets> CD19^-^CD11b^-^CD11c^+^CD69^+^ |
| CD86^+^ cDCs | Live cells>CD45^+^ Singlets> CD19^-^CD11b^-^CD11c^+^CD86^+^ |
| CD54^+^ cDCs | Live cells>CD45^+^ Singlets> CD19^-^CD11b^-^CD11c^+^CD54^+^ |
| B cells | Live cells>CD45^+^ Singlets>CD19^+^ |
| CD69^+^ B cells | Live cells>CD45^+^ Singlets>CD19^+^CD69^+^ |
| CD86^+^ B cells | Live cells>CD45^+^ Singlets>CD19^+^CD86^+^ |
| CD54^+^ B cells | Live cells>CD45^+^ Singlets>CD19^+^CD54^+^ |
| Ki67^+^ B cells | Live cells>CD45^+^ Singlets>CD19^+^Ki67^+^ |
| Ly6C^+^ B cells | Live cells>CD45^+^ Singlets>CD19^+^Ly6C^+^ |
| CD11b^+^ myeloid cells | Singlets>Live cells>CD45^+^CD19^-^CD3^-^CD11b^+^ |
| CD11b^+^ CD69^+^ myeloid cells | Singlets>Live cells>CD45^+^CD19^-^CD3^-^CD11b^+^CD69^+^ |

The following antibodies and gating definitions were used for lymphoid cell immunophenotyping for tDLN and tumor cell samples. Frequencies for each cell subset within their respective parental gate were determined.

Table S3 A: Flow cytometry reagents for analysis of lymphoid cells from mouse tDLN and tumor cell samples

| Marker / Reagent | Fluorochrome | Cat. | Clone | Vendor |
| --- | --- | --- | --- | --- |
| CD62L | PE-Cy7 | 25-0621-82 | MEL-14 | eBioscience |
| CD25 | APC | 17-0251-82 | PC61.5 | eBioscience |
| CD45 | APC-eF780 | 47-0451-82 | 30-F11 | eBioscience |
| CD19 | PE-Cy5 | 115509 | 6D5 | BioLegend |
| CD4 | BV650 | 100469 | GK1.5 | BioLegend |
| CD3e | PerCP-eF710 | 46-0033-82 | eBio500A2 | eBioscience |
| CD8a | AF700 | 56-0081-82 | 53-6.7 | eBioscience |
| CD11b | BV570 | 101233 | M1/70 | BioLegend |
| CD44 | BV785 | 103059 | IM7 | BioLegend |
| ICOS | BV605 | 313537 | C398.4A | BioLegend |
| PD-1 (CD279) | BV711 | 748265 | RMP1-30 | BD |
| NKp46 (CD335) | PE-eF610 | 61-3351-82 | 29A1.4 | eBioscience |
| Ki67 | AF488 | 558616 | B56 | BD |
| FoxP3 | PE | 12-5773-80 | FJK-16s | eBioscience |
| Granzyme B | Pacific Blue | 515408 | GB11 | BioLegend |
| Fc Block (anti-CD16/CD32) | Purified | 14-0161-86 | 93 | eBioscience |
| Live/Dead | eF506 | 65-0866-14 | Not applicable | eBioscience |

Table S3 B: Lymphoid cell populations analyzed in mouse tDLN and tumor cell samples

| Population | Cell Subset Gating Definition |
| --- | --- |
| NK cells | Singlets>Live cells>CD45^+^CD3^-^CD19^-^NKp46^+^ |
| Ki67^+^ NK cells | Singlets>Live cells>CD45^+^CD3^-^CD19^-^NKp46^+^Ki67^+^ |
| Granzyme B^+^ NK cells | Singlets>Live cells>CD45^+^CD3^-^CD19^-^NKp46^+^Granzyme B^+^ |
| CD4^+^ T cells | Singlets>Live cells>CD45^+^CD3^+^CD4^+^ |
| Ki67^+^ CD4^+^ T cells | Singlets>Live cells>CD45^+^CD3^+^CD4^+^Ki67^+^ |
| ICOS^+^ CD4^+^ T cells | Singlets>Live cells>CD45^+^CD3^+^CD4^+^ICOS^+^ |
| CD8^+^ T cells | Singlets>Live cells>CD45^+^CD3^+^CD8^+^ |
| Ki67^+^ CD8^+^ T cells | Singlets>Live cells>CD45^+^CD3^+^CD8^+^Ki67^+^ |
| ICOS^+^ CD8^+^ T cells | Singlets>Live cells>CD45^+^CD3^+^CD8^+^ICOS^+^ |
| Granzyme B^+^ CD8^+^ T cells | Singlets>Live cells>CD45^+^CD3^+^CD8^+^ Granzyme B^+^ |
| PD-1^+^ CD8^+^ T cells | Singlets>Live cells>CD45^+^ CD3^+^CD8^+^PD-1^+^ |
| Ki67^+^ B cells | Singlets>Live cells>CD45^+^CD19^+^ |
| Ki67^+^ B cells | Singlets>Live cells>CD45^+^CD19^+^Ki67^+^ |

The following antibodies and gating definitions were used for tetramer cell immunophenotyping for tDLN and tumor cell samples. Frequencies for each cell subset within their respective parental gate were determined.

Table S4 A: Flow cytometry reagents for analysis of AH1-tetramer^+^ cells from mouse tumor cell samples

| Marker / Reagent | Fluorochrome | Cat. | Clone | Vendor |
| --- | --- | --- | --- | --- |
| CD62L | PE-Cy7 | 25-0621-82 | MEL-14 | eBioscience |
| CD4 | PE-Cy5 | 100514 | RM4-5 | BioLegend |
| CD45 | APC-eF780 | 47-0451-82 | 30-F11 | eBioscience |
| CD19 | AF700 | 115528 | 6D5 | BioLegend |
| CD25 | BV421 | 102034 | PC61 | BioLegend |
| CD11b | BV510 | 101263 | M1/70 | BioLegend |
| CD44 | BV785 | 103059 | IM7 | BioLegend |
| CD3 | PE-eF610 | 61-0032-82 | 17A2 | eBioscience |
| CD69 | BV711 | 104537 | H1.2F3 | BioLegend |
| ICOS | BV605 | 313537 | C398.4A | BioLegend |
| CD8 | FITC | D271-4 | KT15 | MBL International |
| H-2Ld MuLV gp70 Tetramer-SPSYVYHQF | PE | TB-M521-1 | Not applicable | MBL International |
| Live/Dead Fixable Yellow |  | L34968 | Not applicable | Invitrogen |
| Or for a matched control tetramer panel: |  |  |  |  |
| H-2Ld b-galactosidase Tetramer-TPHPARIGL (negative control) | PE | TB-M511-1 | Not applicable | MBL International |

Table S4 B: AH1-tetramer^+^ cell populations analyzed in mouse tumor samples

| Population | Cell Subset Gating Definition |
| --- | --- |
| CD8^+^ T cells | Singlets>Live cells>CD45^+^CD11b^-^CD19^-^CD3^+^CD8^+^ |
| Tetramer^+^ CD8^+^ T cells | Singlets>Live cells>CD45^+^CD11b^-^CD19^-^CD3^+^CD8^+^AH1-Tetramer^+^ |

***Additional ARRIVE (Animal Research: Reporting of In Vivo Experiments) details for mouse experiments***

Mouse tumor experiments were performed inoculating approximately double the number of mice to be enrolled in single tumor bearing studies, and 3-4 times the number of mice to be enrolled in dual-tumor bearing studies, were inoculated in order to meet pre-dose target tumor volume average and range (as indicated in figure legends). Mice were randomized and enrolled into the study using either a stratified randomization method (Figures 1A and Supplemental Figures 2 and 3, Charles River, proprietary software) or using a Matched Distribution method (all other tumor studies, StudyLog™ software). 9–20 animals per treatment condition were used to assess efficacy and enable sample acquisition at indicated timepoints in order to overcome the expected variability in syngeneic mouse tumor studies while still minimizing the number of animals used. To minimize potential confounding effects, treatments with similar agents were performed together, measurements for tumor volumes and body weights were performed in a consistent order (by treatment group), and animals were all housed in the same room. The studies were not run in a blinded fashion.

CT26 experiments used either naïve healthy female BALB/c mice sourced by either Charles River Discovery Research Services Germany GmbH (Freiburg, BW, Germany), Charles River Discovery Services (Morrisville, NC, USA), Crown Bioscience Inc. (Taicang, JS, China), or purchased from Taconic Biosciences (USA). MC38 experiments used naïve healthy female C57BL/6 mice sourced by Charles River Discovery Services (Morrisville, NC, USA). PK experiments used naïve healthy male WISTAR rats sourced by Heidelberg Pharma AG (Ladenburg, BW, Germany).

Mouse work was performed either at Charles River Discovery Research Services Germany GmbH (Freiburg, BW, Germany), Charles River Discovery Services (Morrisville, NC, USA), Crown Bioscience Inc. (Taicang, JS, China), or Ascendis Inc., Redwood City CA (California Department of Public Health Certificate 081). Rat work was performed at Heidelberg Pharma AG (Ladenburg, BW, Germany). Mouse work at Charles River Discovery Research Services Germany GmbH was approved by local animal welfare authorities (study references P646B S209, P646B3 S215, and P646D S249; approval reference G-17/78) and performed in accordance with the German Animal Welfare Act and with the regulations of the Association for Assessment and Accreditation of Laboratory Animal Care (AAALAC). Mouse work at Charles River Discovery Services was approved by a local Institutional Animal Care and Use Committee (IACUC) (study reference MC38-e462; approval reference ASP #980702) and performed in accordance with The Guide for the Care and use of Laboratory Animals and the US Public Health Service (PHS) Policy on Humane Care and Use of Laboratory Animals. The site is AAALAC accredited and has an Office of Laboratory Animal Welfare (OLAW) assurance. Mouse work at Crown Bioscience Inc. was approved by the Crown Bioscience Inc. IACUC (study reference E4649-U1904 and E4649-U1920; approval references AN-1903-05-1067 and AN-1903-05-1067, respectively) and performed in accordance with the regulations of the AAALAC. Rat work at Heidelberg Pharma AG was approved by local animal welfare authorities and performed in accordance with the German Animal Welfare Act. Mouse work at Ascendis Pharma was reviewed and approved by the Ascendis Inc. Redwood City IACUC (study references VIV-M-006, VIV-M-030, and VIV-M-032; approval reference 20200507-ASN-02) and performed in accordance with The Guide for the Care and use of Laboratory Animals, the PHS Policy on Humane Care and Use of Laboratory Animals, and the USDA Animal Welfare Act.
