# Supplementary figures and images for: Intratumoral delivery of TransCon™ TLR7/8 Agonist promotes sustained anti-tumor activity and local immune cell activation while minimizing systemic cytokine induction
Source: Cancer Cell Int. 2022 Sep 19;22:286. doi: 10.1186/s12935-022-02708-6 (PMC9484246; doi:10.1186/s12935-022-02708-6)

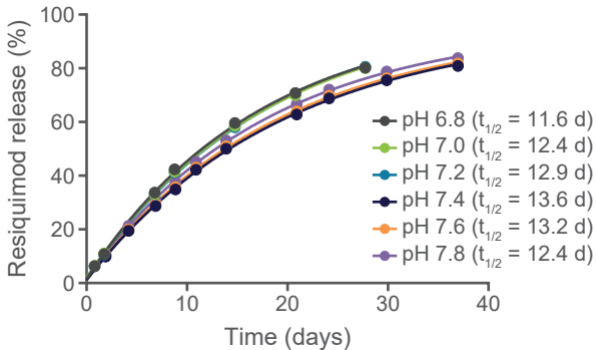

Supplement: Supplementary file 2 — Additional file 2: Figure S1. Consistent resiquimod release in vitro from TransCon TLR7/8 Agonist in the pH range between 6.8 and 7.8. A suspension of TransCon TLR7/8 Agonist (nominal 47 μg eq. of resiquimod) in 60 mM phosphate buffer was incubated at 37 °C at various pH levels. Samples of the supernatant were withdrawn at various times and the resiquimod content was determined. Values are represented as mean percentage from 2 experiments of resiquimod release of total resiquimod loaded on the hydrogel. Release half-lives were determined following a first-order fit of the data. [file 12935_2022_2708_MOESM2_ESM.pdf]

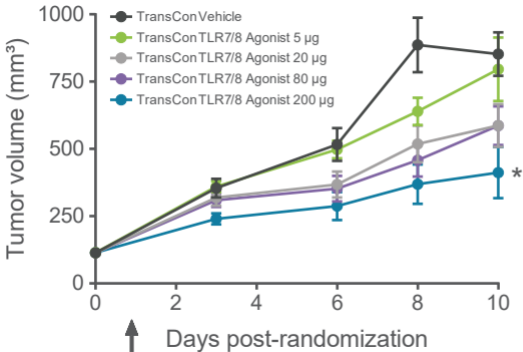

Supplement: Supplementary file 3 — Additional file 3: Figure S2. TransCon TLR7/8 Agonist inhibited CT26 tumor growth in a dose-dependent manner. Female BALB/c mice were SC implanted with 3 x 10^5^ CT26 tumor cells in their flank. When tumors were grown to a mean tumor volume of ~115 mm3, mice were randomized into treatment cohorts (Day 0; n = 17/group). The day following randomization, animals received either empty hydrogel (TransCon Vehicle) or 5, 20, 80, or 200 μg (eq. of resiquimod) of TransCon TLR7/8 Agonist as a single intratumoral dose (arrow). Values are represented as mean tumor volume ± SEM. On Day 10, * = p<0.05 vs TransCon Vehicle, n = 7-8/group. [file 12935_2022_2708_MOESM3_ESM.pdf]

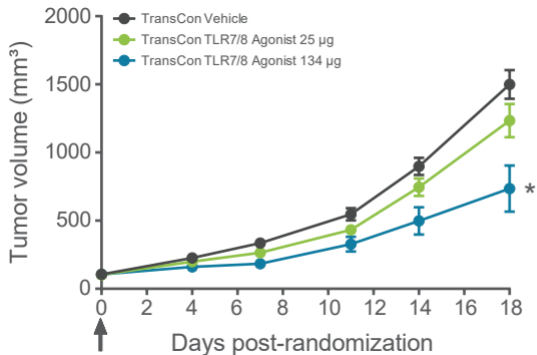

Supplement: Supplementary file 4 — Additional file 4: Figure S3. TransCon TLR7/8 Agonist inhibited MC38 tumor growth in a dose-dependent manner. Female C57BL/6 mice were SC implanted with 5 x 10^5^ CT26 tumor cells in their flank. When tumors were grown to a mean tumor volume of ~100 mm3, mice were randomized into treatment cohorts (Day 0; n = 20-22/group). On the same day of randomization, animals received either empty hydrogel (TransCon Vehicle) or 25 or 134 μg (eq. of resiquimod) of TransCon TLR7/8 Agonist as a single intratumoral dose (arrow). Values are represented as mean tumor volume ± SEM. On Day 18, * = p<0.05 vs TransCon Vehicle, n = 13-17/group. [file 12935_2022_2708_MOESM4_ESM.pdf]
